# Analysis of Chloroplast Genomes Provides Insights Into the Evolution of *Agropyron*

**DOI:** 10.3389/fgene.2022.832809

**Published:** 2022-01-25

**Authors:** Huijie Han, Rui Qiu, Yefei Liu, Xinyue Zhou, Cuiping Gao, Yongzhen Pang, Yan Zhao

**Affiliations:** ^1^ Key Laboratory of Grassland Resources (IMAU), Key Laboratory of Forage Cultivation, Processing and High Efficient Utilization, College of Grassland, Resource and Environmental Science, Ministry of Education, Ministry of Agriculture, Inner Mongolia Agricultural University, Hohhot, China; ^2^ Institute of Animal Science, The Chinese Academy of Agricultural Sciences, Beijing, China

**Keywords:** *Agropyron*, chloroplast genome, phylogenetic relationship, comparative analysis, illumina sequencing

## Abstract

Plants of the *Agropyron* genus are important pasture resources, and they also play important roles in the ecological restoration. Chloroplast genomes are inherited from maternal parents, and they are important for studying species taxonomy and evolution. In this study, we sequenced the complete chloroplast genomes of five typical species of the *Agropyron* genus (eg., *A. cristatum* × *A. desertorum* Fisch. Schult, *A. desertorum*, *A. desertorum* Fisch. Schult. cv. Nordan, *A. michnoi* Roshev, and *A. mongolicum* Keng) using the Illumina NovaSeq platform. We found that these five chloroplast genomes exhibit a typical quadripartite structure with a conserved genome arrangement and structure. Their chloroplast genomes contain the large single-copy regions (LSC, 79,613 bp-79,634 bp), the small single-copy regions (SSC, 12,760 bp-12,768 bp), and the inverted repeat regions (IR, 43,060 bp-43,090 bp). Each of the five chloroplast genomes contains 129 genes, including 38 tRNA genes, eight rRNA genes, and 83 protein-coding genes. Among them, the genes *trnG-GCC*, *matK*, *petL*, *ccsA*, and *rpl32* showed significant nucleotide diversity in these five species, and they may be used as molecular markers in taxonomic studies. Phylogenetic analysis showed that *A. mongolicum* is closely related to *A. michnoi*, while others have a closer genetic relationship with the *Triticum* genus.

## Introduction

The *Agropyron* genus contains many important wild species, which is a close relative of the *Triticum* genus. Different species of the *Agropyron* genus have distinct chromosome ploidies, including diploid, PP, 2n = 2x = 14; tetraploid, PPPP, 2n = 4x = 28; and hexaploid, PPPPPP, 2n = 6x = 42 (Dewey, 1984; Fordlloyd et al., 2011). Most species of the *Agropyron* genus are excellent sources of forage and habitat for livestock and wildlife. They are also valuable for weed control, habitat use, soil stabilization, and watershed management (Wang, 2011). Species of *Agropyron* also possess a lot of valuable traits, such as tolerance to drought and cold, resistance to diseases, and high yield (Asay et al., 1985; Dong et al., 1992; Lu et al., 2015). They are high-quality forage for grassland improvement and valuable genetic resources for wheat improvement (Ochoa et al., 2015; Sun et al., 2021).

The *Agropyron* genus contains several major species, namely, *A. cristatum*, *A. mongolicum*, *A. desertorum*, *A. michnoi*, and *A. cristatum* × *A. desertorum* Fisch. Schult. Among them, *A. cristatum* is originated from Iran and distributed in arid, semi-arid, and alpine regions (Dewey, 1984). *A. mongolicum*, a unique species in China, is mainly distributed in Shaanxi, Inner Mongolia, Gansu, Ningxia, Shanxi, and other areas of China (Guo, 1987). Both *A. cristatum* and *A. mongolicum* have a diploidy group, but these two species are distinct in general morphology (Dewey, 1984; Dewey,1981). In traditional taxonomy, the species of *Agropyron* is mainly recognized by the spike morphology. *A. mongolicum* differs from *A. cristatum* by its narrow, linear spikes. All other diploid accessions within the *Agropyron* genus are similar to *A. cristatum* with broad spikes. These two species could hybridize easily, and the F1 hybrids displayed better characteristics (Dewey, 1984; Hsiao et al., 1986). Among the species of the *Agropyron* genus distributed in China, *A. cristatum* and *A. mongolicum* are genetically differentiated species, while *A. desertorum* is the intermediate one. *A. desertorum*, *A. mongolicum*, and *A. michnoi* are the offspring species of *A. cristatum* that share the same basic genome from the counterpart based on SSR analysis (Che et al., 2015). *A. cristatum* × *A. desertorum* Fisch. Schult. is a distant hybrid of the induced tetraploid *A. cristatum* and the natural tetraploid *A. desertorum* (Dewey and Pendse, 1968; Asay and Johnson, 1990).

Chloroplast is a vital organelle in plants, which plays an important role in photosynthesis. Chloroplast is involved in plant physiology and development, the biosynthesis of amino acids, nucleotides, fatty acids, phytohormones, vitamins, and the assimilation of sulfur and nitrogen (Daniell et al., 2016a). The chloroplast genome contains genes with functions in many processes of photosynthesis and other metabolism. The chloroplast genome possesses highly conserved DNA sequences at a low substitution level (especially in inverted repeat regions). Therefore, the chloroplast genome is ideal for phylogenetic inference at species and higher orders. The chloroplast genome makes a large contribution to the search of phylogeny in the plant family and solves the group evolution relationship from phylogeny (Daniell et al., 2016a). Similarly, it is essential to study the chloroplast genome of the *Agropyron* genus for a better understanding on its classification, phylogenetic relationship, and variation among species.

In this study, in order to provide comprehensive insights into the evolution of the chloroplast genomes of the *Agropyron* genus, we sequenced the chloroplast genomes of five *Agropyron* species. In addition, we conducted comparative analyses on the chloroplast genome of five *Agropyron* species and eleven other related species. Therefore, our study provides valuable information for the phylogenetic relationship and species identification within the *Agropyron* genus.

## Materials and Methods

### Sampling, Chloroplast DNA Extraction, and Sequencing

*Agropyron* plants were grown at the pasture experimental station of the Inner Mongolia Agricultural University, located in Hohhot, Inner Mongolia Autonomous Region. The experimental station had a typical temperate continental climate with precipitation and heat in the same season. The average annual temperature was 8°C, and the frost-free period was 130–140 d. The soil was sandy loam, with medium fertility and a pH of 7.0–7.5.

Fresh leaves were collected and snap-frozen in liquid nitrogen and then stored at−80°C until DNA extraction. DNA extraction was performed using the modified CTAB method (Doyle, 1987). DNA quality was assessed using a nanodrop spectrophotometer. Genomic DNA samples were mechanically fragmented, and then, fragments were detected by agarose gel electrophoresis. The library for sequencing was amplified by PCR, and sequencing was performed using an Illumina NovaSeq platform with PE150 based on sequencing by synthesis (SBS).

### Chloroplast Genome Annotation

Fastp (version 0.20.0, https://github.com/OpenGene/fastp) software was used to filter the raw data by removing the joint sequences and low-quality reads. High-quality clean data were assembled according to chloroplast genome sequences of reference species to obtain chloroplast sequence assembly results (Langmead and Salzberg, 2012). Two methods were used to annotate chloroplast genomes to improve the accuracy. First, the Prodigal v2.6.3 was used for annotation of chloroplast CDS (https://www.github.com/hyattpd/Prodigal), and the hmmer v3.1 b2 (http://www.hmmer.org/) software and aragorn v1.2.38 (http://130.235.244.92/ARAGORN/) were used to predict rRNA and tRNA. Second, based on the gene sequences of available related species in the NCBI, gene sequences of *Agropyron* were extracted, and then, BLAST v2.6 (https://blast.ncbi.nlm.nih.gov/Blast. cgi) was used to obtain the second round annotation. Then, different genes from the two annotation results were manually checked, the wrong annotations and redundant annotations were removed, and the multi-exon boundary and the final annotation were accomplished. The final step was carried out by Gene Pioneer Biotechnologies (Nanjing, China).

### Analysis of the Ka/Ks Ratio

The Ka/Ks ratio is a reflection of selection pressures. Mafft v7.310 (https://mafft.cbrc jp/alignment/software/) was used for the sequence alignment, and the software KaKs Calculator v2.0 (https://sourceforge.net/projects/kakscalculator2/) was used to calculate Ka and Ks values (Zhang et al., 2006).

### Analyses of Dispersed Repeats and SSR

Vmatch V2.3.0 (http://www.vmatch.de/) software was used in combination with Perl scripts to identify duplicate sequences. The parameters were set as: minimum length = 30 bp hamming distance = 3, and the identification was in four forms: forward, palindromic, reverse, and complement. Simple sequence repeats (SSRs) are a class of markers containing several nucleotides (usually one–six) as repeats. SSR markers in chloroplast genomes are called cpSSR markers. We used the software MISA v1.0 (MIcroSAtellite identification tool, http://pgrc.ipk-gatersleben.de/misa/misa.html) for cpSSR analysis.

### Analysis of Sequence Variation

Mafft software (Auto mode) was used for global alignment of homologous gene sequences in different species. MEGA X (Kumar et al., 2008) was used to calculate the level of SNV (single nucleotide variation) and the average genetic distance of chloroplast genome sequences. DnaSP v6 (Julio) was used to determine the nucleotide diversity with parameter settings as follows: a step length of 200 bp and window length of 800 bp (Rozas et al., 2003).

### Comparison of the IR/SC Boundary

SC/IR boundary data were calculated based on the annotated information of the chloroplast genomes of five species of *Agropyron*, and the simple chloroplast genome structure diagrams of five species were drawn according to the statistical results (Daniell et al., 2016b). The boundary information of the four regions of chloroplast genomes of the *Agropyron* was compared, and the contraction and expansion of IR regions were observed.

### Analysis on the Phylogenetic Relationship

A genome-wide evolutionary tree was constructed. The ring sequence was analyzed with the same starting point, and the full-length sequences were used to carry out the multiple sequence alignment with MAFFT software (v7.427, - auto mode), followed by correction with trim Al (v1.4. Rev15). By using the software RAxML v8.2.10 (https://cme.h-its.org/exelixis/software.html) and choosing the GTRGAMMA model with a rapid bootstrap analysis, a maximum likelihood evolutionary tree was constructed with a bootstrap of 1,000 (Katoh et al., 2005).

## Results

### Phenotypic Identification of the Six *Agropyron* Species

**FIGURE 1:**
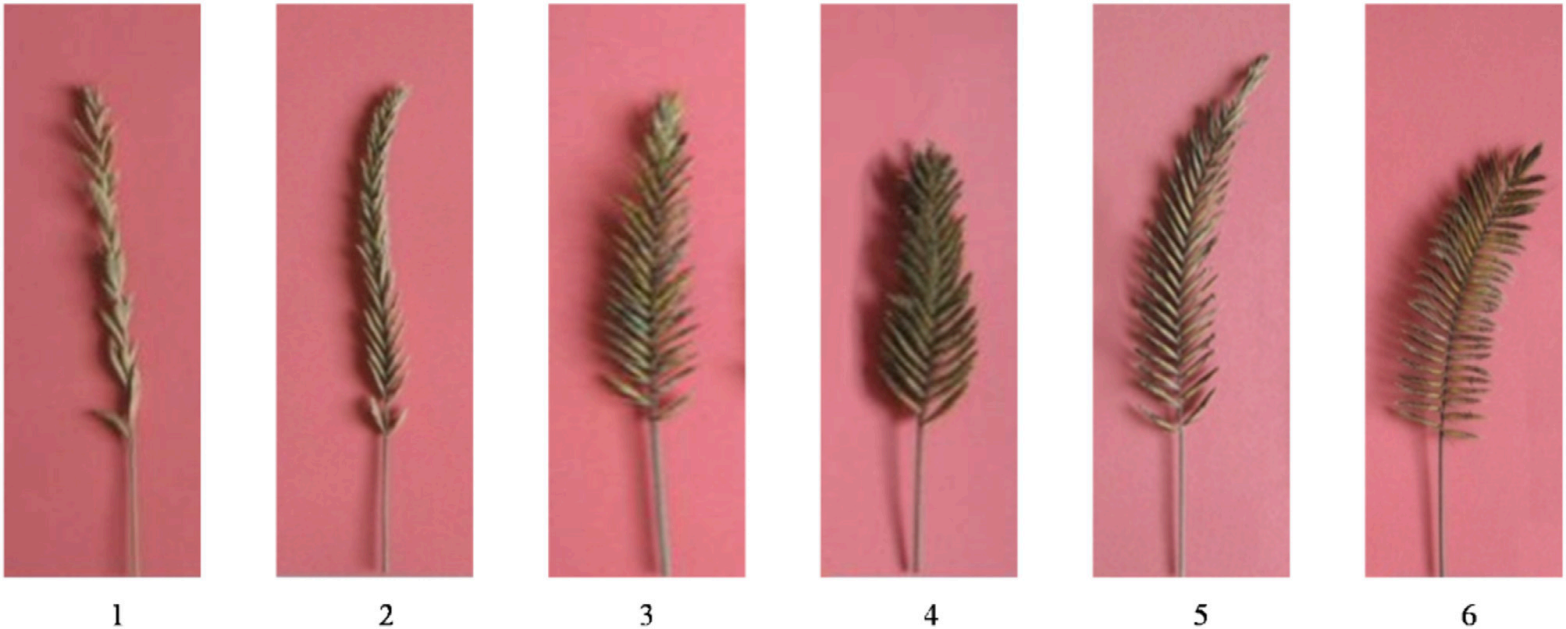
Phenotype of the spikelet of six *Agropyron* species as numbered in Table 1.

Among all species investigated in this study, *A. mongolicum* Keng, *A. desertorum*, *A. michnoi*, and *A. cristatum* are widely distributed in China (Table 1). *A. cristatum* × *A. desertorum* Fisch. Schult and *A. desertorum* Fisch. Schult. have been used in the construction of artificial grasslands in northern China. The angles between spikelets and cobs are different among the six species, with the angle gradually increased in an order of *A. mongolicum*, *A. desertorum* Fisch. Schult, *A. desertorum*, *A. michnoi*, *A. cristatum*× *A. desertorum* Fisch. Schult, and *A. cristatum* (Figure 1). The smallest angle is found in *A. mongolicum*, whereas the largest angle, which is close to right angle, is found in *A. cristatum*. The spikelet of *A. mongolicum* is long, thin, and sparsely arranged. The spikelets of *A. michnoi* and *A. cristatum* are short. The spikelet of *A. cristatum*× *A. desertorum* Fisch. Schult. is the biggest (Figure 1). Although they showed different phenotypes, the phenological periods of the six *Agropyron* species are basically the same, and they could be crossed among each other in nature, except for *A. mongolicum*.

**TABLE 1:** Genotypes and phenotypes of six *Agropyron* species.

Species	Genome	Ploidy	Origin
1: *A. mongolicum* Keng	PP	Diploid	China
2: *A. desertorum* Fisch.Schult. cv. Nordan	PPPP	Tetraploid	United States
3: *A. desertorum*	PPPP	Tetraploid	China
4: *A. michnoi* Roshev	PPPP	Tetraploid	China
5: *A. cristatum* L. Gaertn.×*A. desertorum* Fisch. Schult	PPPP	Tetraploid	United States
6: *A. cristatum* L.	PP/PPPP	Diploid/Tetraploid	China

### Features of the Chloroplast Genomes in *Agropyron*

The sizes of the chloroplast genome of *A. cristatum*× *A. desertorum* Fisch. Schult, *A. desertorum*, *A. desertorum* Fisch. Schult, *A. michnoi*, and *A. mongolicum* are 135,459 bp, 135,455 bp, 135,461 bp, 135,455 bp, and 135,486 bp in length, respectively. We also compared them with the available chloroplast genome of *A. cristatum* (KY126307.1) (Chen et al., 2018), which is 135,554 bp in length. Our results showed that the lengths of these five *Agropyron* species are similar.

Each of the five genomes contains 129 genes, including 38 tRNA genes, eight rRNA genes, and 83 protein-coding genes. As a comparison, the gene number of *A. cristatum* chloroplast genome (KY126307.1) is different from other *Agropyron* species, which is short and contains only 109 genes, including 29 tRNA genes, four rRNA genes, and 76 protein-coding genes (Chen et al., 2018) (Tables 2, 3). The GC contents of *A. cristatum* × *A. desertorum* Fisch. Schult, and *A. desertorum* are exactly the same (38.32%), and the GC contents of the other four *Agropyron* species are the same (Table 2, Supplementary Tables S1–S5).

**TABLE 2:** Comparison of the chloroplast genome features of five *Agropyron*.

Species	Genome Size (bp)	LSC length (bp) (GC content)	SSC length (bp) (GC content)	IR length (bp) (GC content)	Gene	tRNA	rRNA	Protein-coding gene	GC content (%)
*A. cristatum* L. Gaertn. × *A. desertorum* Fisch. Schult.	135,459	79,617 (36.27%)	12,760 (32.24%)	43,082 (43.91%)	129	38	8	83	38.32
*A. desertorum*	135,455	79,613 (36.28%)	12,760 (32.23%)	43,082 (43.92%)	129	38	8	83	38.32
*A. desertorum* (Fisch.) Schult. cv. Nordan	135,461	79,620 (36.28%)	12,761 (33.22%)	43,080 (43.91%)	129	38	8	83	38.33
*A. michnoi* Roshev	135,455	79,627 (36.29%)	12,768 (32.23%)	43,060 (43.92%)	129	38	8	83	38.33
*A. mongolicum* Keng	135,486	79,634 (36.29%)	12,762 (32.24%)	43,090 (43.91%)	129	38	8	83	38.33
*A. cristatum* (L.) Gaertn. (KY126307.1)	135,554	79,623 (36.28%)	12,769 (32.29%)	21,581 (43.89%)	109	29	4	76	38.33

**TABLE 3:** List of genes present in the *Agropyron* chloroplast genome

Category	Gene group	Gene name
Photosynthesis	Subunits of photosystem I	*psaA, psaB,psaC, psaI,psaJ*
	Subunits of photosystem II	*psbA, psbB,psbC, psbD,psbE, psbF,psbH, psbI,psbJ, psbK,psbL, psbM,psbN, psbT,psbZ*
	Subunits of NADH dehydrogenase	ndhA*,ndhB*(2),ndhC, ndhD,ndhE, ndhF,ndhG, ndhH,ndhI, ndhJ,ndhK
	Subunits of the cytochrome b/f complex	petA, petB*,petD*,petG, petL,petN
	Subunits of ATP synthase	atpA, atpB,atpE, atpF*,atpH, atpI
	Large subunit of rubisco	*rbcL*
	Subunits photochlorophyllide reductase	*-*
Self-replication	Proteins of the large ribosomal subunit	rpl14,rpl16*,rpl2*(2),rpl20,rpl22,rpl23(2),rpl32,rpl33,rpl36
	Proteins of the small ribosomal subunit	rps11,rps12**(2),rps14,rps15(2),rps16*,rps18,rps19(2),rps2,rps3,rps4,rps7(2),rps8
	Subunits of RNA polymerase	*rpoA, rpoB,rpoC1,rpoC2*
	Ribosomal RNAs	*rrn16(2),rrn23(2),rrn4.5(2),rrn5(2)*
	Transfer RNAs	trnA-UGC*(2),trnC-GCA,trnD-GUC,trnE-UUC,trnF-GAA,trnG-GCC*,trnG-UCC,trnH-GUG(2),trnI-CAU(2),trnI-GAU*(2),trnK-UUU*,trnL-CAA(2),trnL-UAA*,trnL-UAG,trnM-CAU,trnN-GUU(2),trnP-UGG,trnQ-UUG,trnR-ACG(2),trnR-UCU,trnS-GCU,trnS-GGA,trnS-UGA,trnT-GGU,trnT-UGU,trnV-GAC(2),trnV-UAC*,trnW-CCA,trnY-GUA,trnfM-CAU
Other genes	Maturase	*matK*
	Protease	*clpP*
	Envelope membrane protein	*cemA*
	Acetyl-CoA carboxylase	*-*
	c-type cytochrome synthesis gene	*ccsA*
	Translation initiation factor	*infA*
	Other	*-*
Genes of unknown function	Conserved hypothetical chloroplast ORF	ycf3**,ycf4

a Notes: Gene*: gene with one introns; Gene**: gene with two introns; #Gene: pseudo gene; Gene (2): number of copies of multi-copy genes.

**FIGURE 2:**
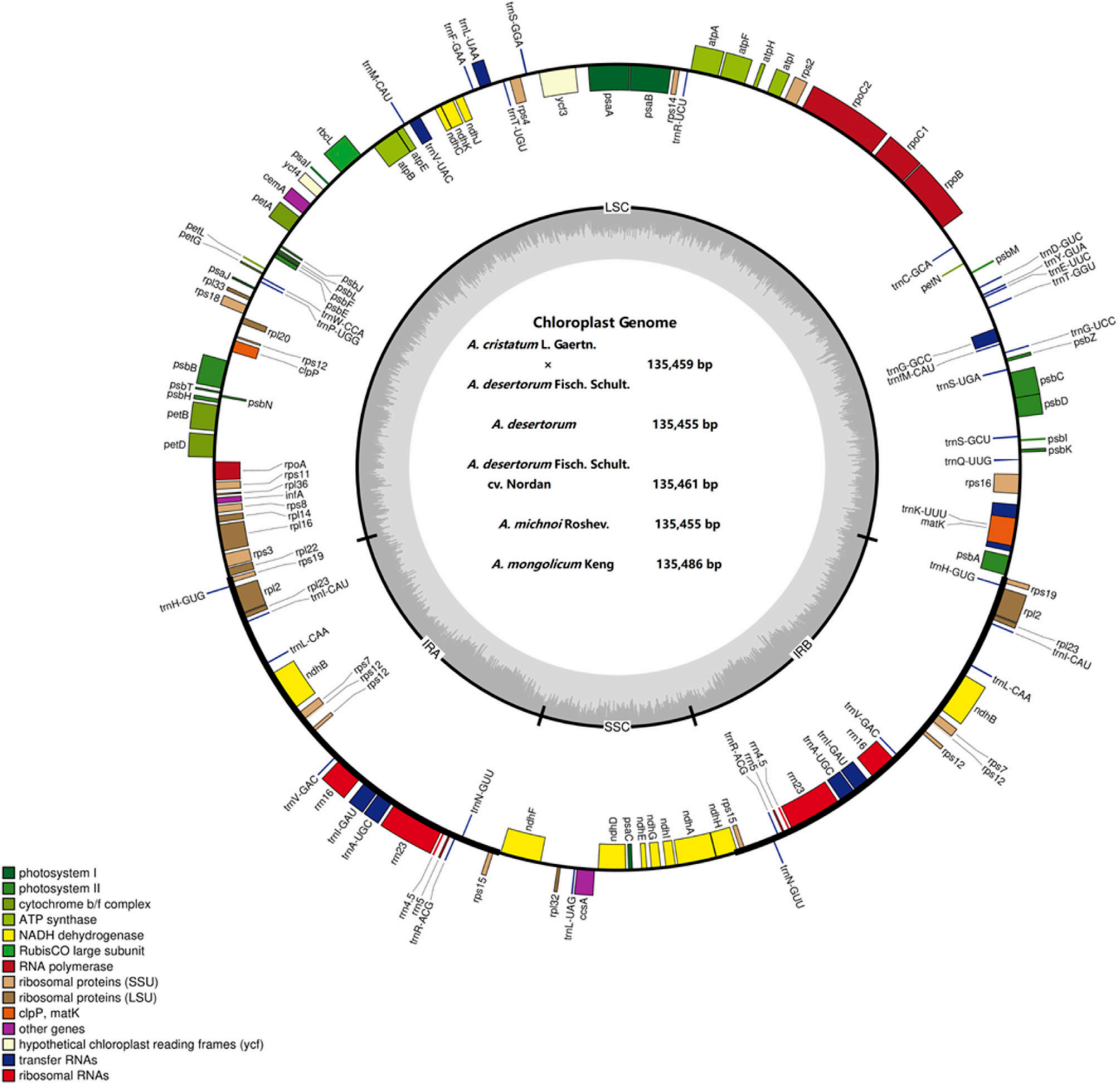
Representative map of the *Agropyron* chloroplast genome. The plus encoding gene is on the outside of the circle, and the minus encoding gene is on the inside of the circle. The inner gray circle represents the GC content.

The complete chloroplast genomes of the *Agropyron* species exhibit a typical single-circular molecule with a four-segmented structure (Figure 2), consisting of a large single-copy region (LSC), a small single-copy region (SSC), and the inverted repeat regions a and b (IRa and IRb), which is similar to many other plant species (Pasquale et al., 2015; Qiu et al., 2019). In every *Agropyron* chloroplast genome, the IR region contains eight rRNA genes, 16 tRNA genes, and 17 protein-coding genes; the SSC region contains one tRNA gene and 10 protein-coding genes, while the LSC region contains 21 tRNA genes and 56 protein-coding genes. Though the GC contents of the complete chloroplast genome are similar in different *Agropyron* species (38.32–38.33%, Table 2), the GC contents in IR regions (43.89–43.92%) are significantly higher than those in the SSC region (36.27–36.29%) and LSC region (32.23–33.22%), and this pattern is consistent with these five *Agropyron* genomes (Table 2).

### Analysis of the Ka/Ks Ratio

**FIGURE 3:**
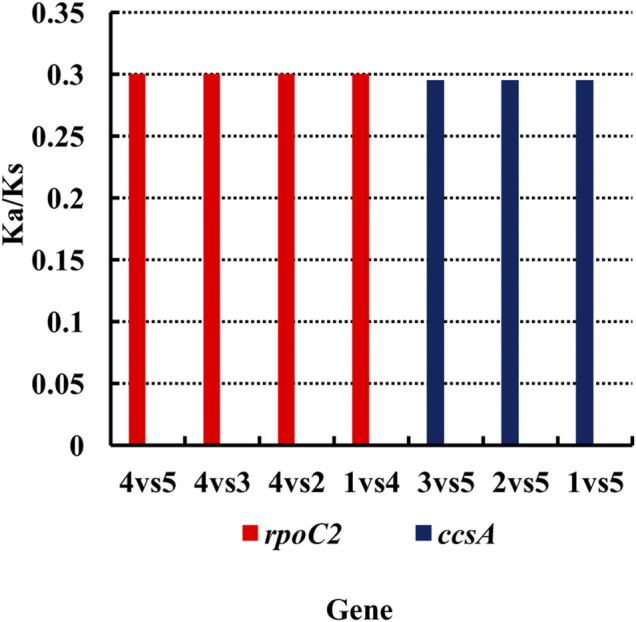
Analysis of the Ka/Ks ratio of *Agropyron*. *X*-axis: name of the gene. *Y*-axis: value of Ka/Ks. 1–5: *A. cristatum* × *A. desertorum* Fisch. Schult, *A. desertorum*, *A. desertorum* Fisch. Schult, *A. michnoi*, and *A. mongolicum*. The red bars indicate the *rpoC2* gene, and the blue bars indicate the *ccsA* gene.

Nucleotide substitutions may or may not lead to changes in amino acids. If the substitution does not cause an amino acid change, then it is a synonymous mutation, but if the substitution does cause an amino acid change, it is a non-synonymous mutation. Non-synonymous mutations in general are the forces of natural selection. The ratio of the non-synonymous mutation rate (Ka) to synonymous mutation rate (Ks) is used to determine the selection outcomes. If the ratio is greater than 1, it indicates a positive selection. If the ratio is less than 1, it indicates a purifying selection. Therefore, we analyzed the Ka and Ks values for each genes of the chloroplast genome and compared the Ka/Ks ratios among these six species of *Agropyron* (Supplementary Tables S6–15). It showed that these species do not have obvious nucleotide changes for almost all the genes without available Ka/Ks values (Supplementary Tables S6–15). However, the Ka/Ks values for the *ropC*2 gene in *A. michnoi* and the *ccsA* gene in *A. mongolicum* were relatively high when compared with other species (Figure 3, Supplementary Tables S6–12), indicating these two genes contained obvious mutations during evolution.

### Analysis of Dispersed Repeats and SSR

**FIGURE 4:**
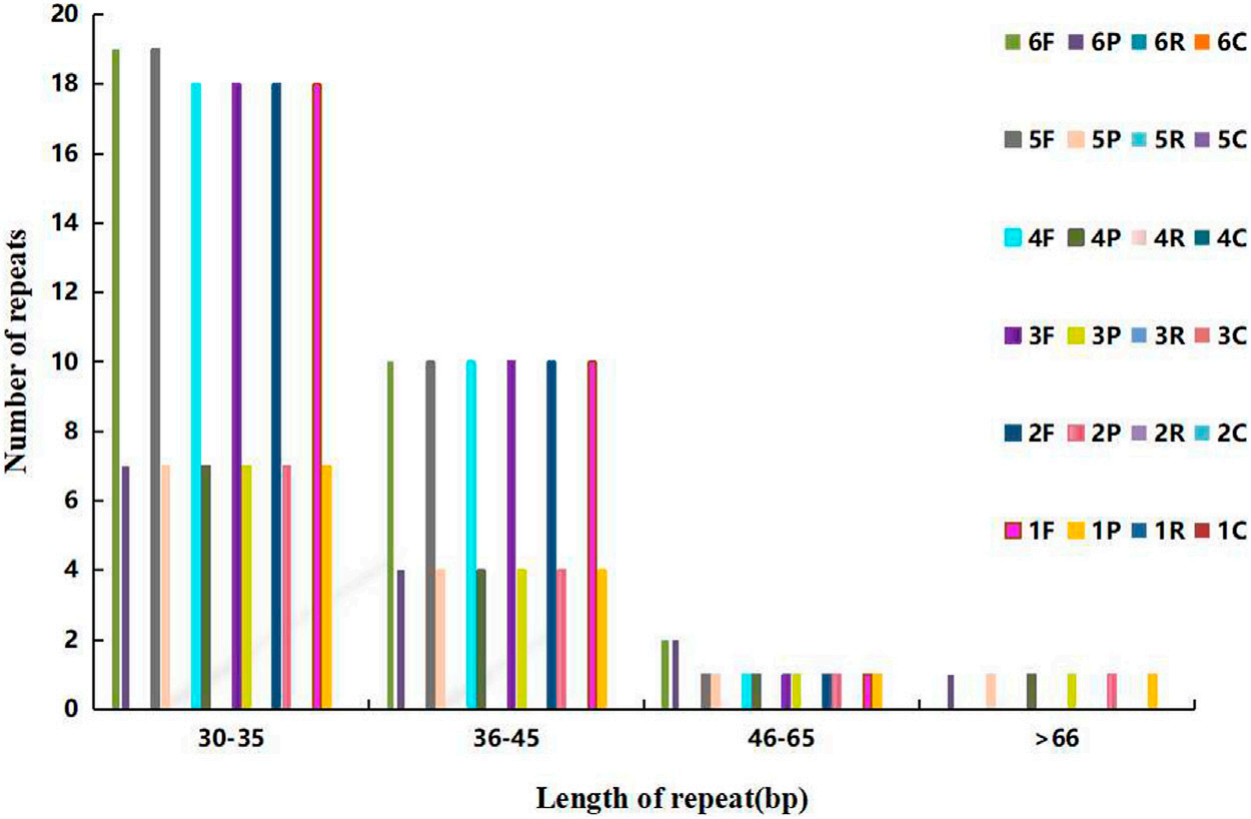
Distributions of the dispersed repeat. The x-coordinate is the length of dispersed repeats, and the y-coordinate is the number of dispersed repeats. 1–6: *A. cristatum*× *A. desertorum* Fisch. Schult, *A. desertorum*, *A. desertorum* Fisch. Schult, *A. michnoi*, *A. mongolicum*, and *A. cristatum*. F stands for forward repeats, P for palindromic repeats, R for reverse repeats, and C for complementary repeats.

The dispersed repeats of *Agropyron* are similar except for *A. mongolicum* and *A. cristatum*, in which the forward repeats of 30–35 bp are different from that in other *Agropyron* chloroplast genomes. Different from other species, chloroplast genomes of *Agropyron* have no reverse or complementary repeats (Figure 4). There are more forward repeats than palindromic repeats in the chloroplast genomes of *Agropyron*.

**FIGURE 5:**
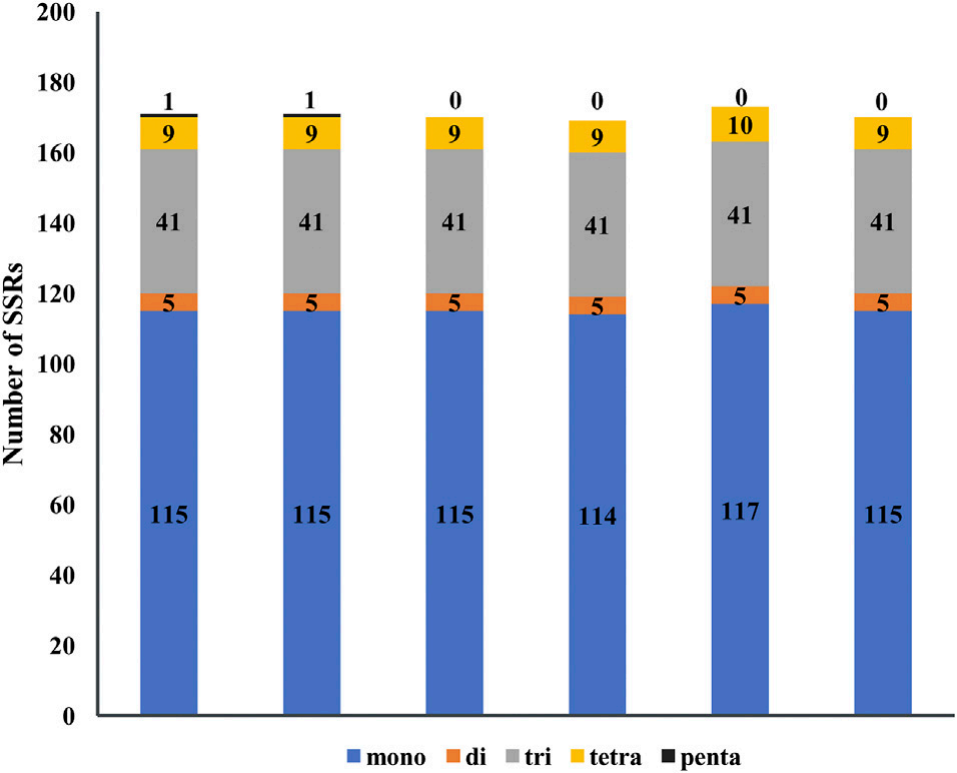
Analysis of simple sequence repeats in the chloroplast genome of six *Agropyron* species. Numbers indicate SSR types detected in each species. *X*-axis: name of the species. *Y*-axis: number of SSR.

Simple sequence repeats (SSRs) consist of six or less than six base repeats and distribute widely in chloroplast genomes, and mono-, di-, tri-, tetra-, and penta-nucleotide repeats are found in five species of *Agropyron*. In total, 171, 171, 170, 169, 173, and 169 SSRs were found in *A. cristatum* × *A. desertorum* Fisch. Schult, *A. desertorum*, *A. desertorum* Fisch. Schult, *A. michnoi*, *A. mongolicum*, and *A. cristatum*, respectively (Figure 5). The number of mononucleotide repeats is the largest, similar as observed in other plant species. The mono-nucleotide and tetra-nucleotide SSRs in *A. mongolicum* are slightly higher than other species. Penta-nucleotide SSRs are only found in *A. cristatum* × *A. desertorum* Fisch. Schult. and *A. desertorum* (Figure 5). Most mono-nucleotide SSRs are polyadenine (poly A) or polythymine (poly T), accounting for about 95%. The result also shows that *A. cristatum* × *A. desertorum* Fisch. Schult. is the closest species to *A. desertorum* from the quantity and length of repeated sequences.

### Analysis on Sequence Variation

**FIGURE 6:**
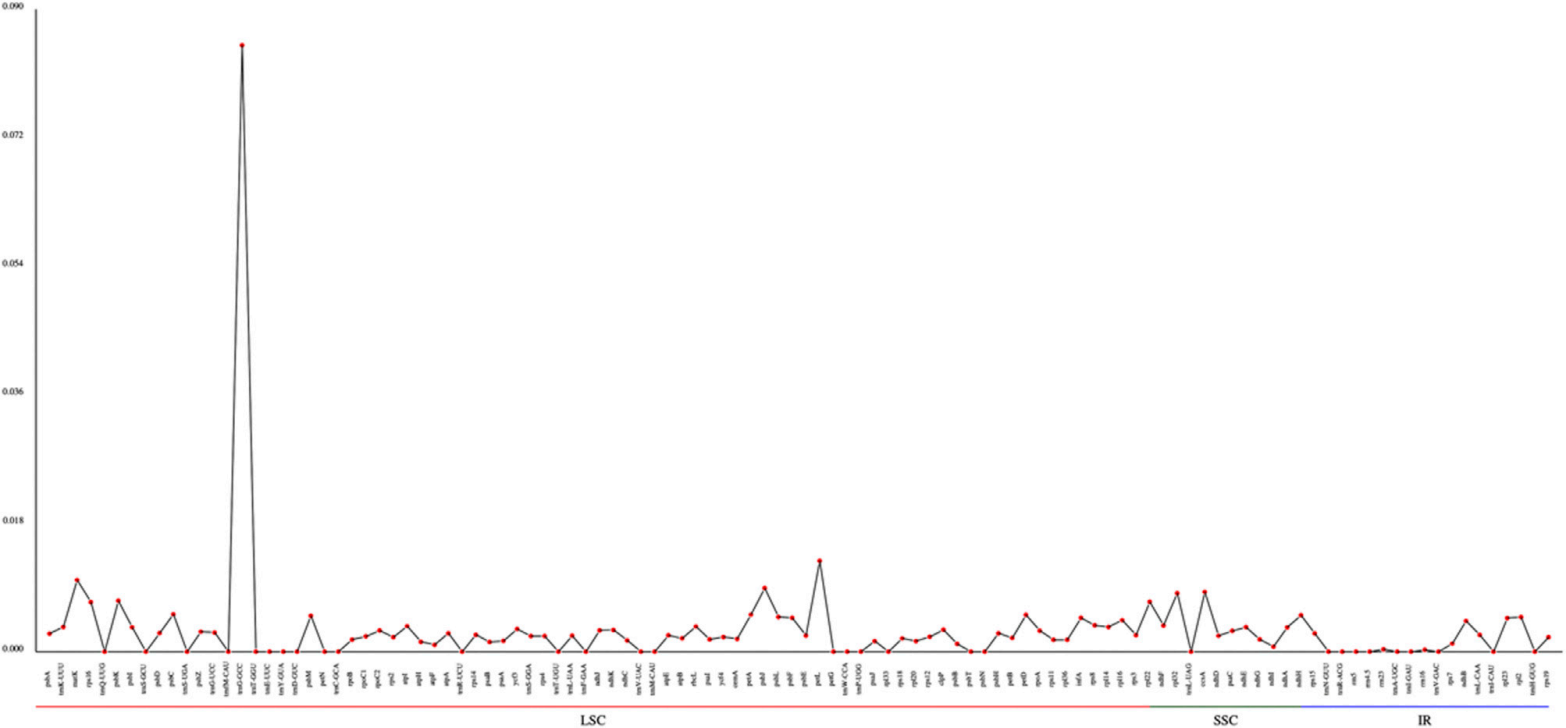
Line graph showing the gene PI value. *X*-axis: name of the gene. *Y*-axis: value of PI.

The Pi values (nucleotide diversity) can reveal the variation size of the nucleic acid sequence in different species, and regions with high degree of variations can be used as potential molecular markers for population genetics. Using the Mafft software, we performed global alignment of homologous genes in different species. Our results showed that the *trnG-GCC* gene in the LSC region has the largest diversity, with the maximum value of 0.0849. The differences of nucleotide diversity in other regions are not obvious. The nucleotide diversity in LSCs and SSCs is substantially higher than that in IRs (Figure 6, Supplementary Table S17).

**FIGURE 7:**
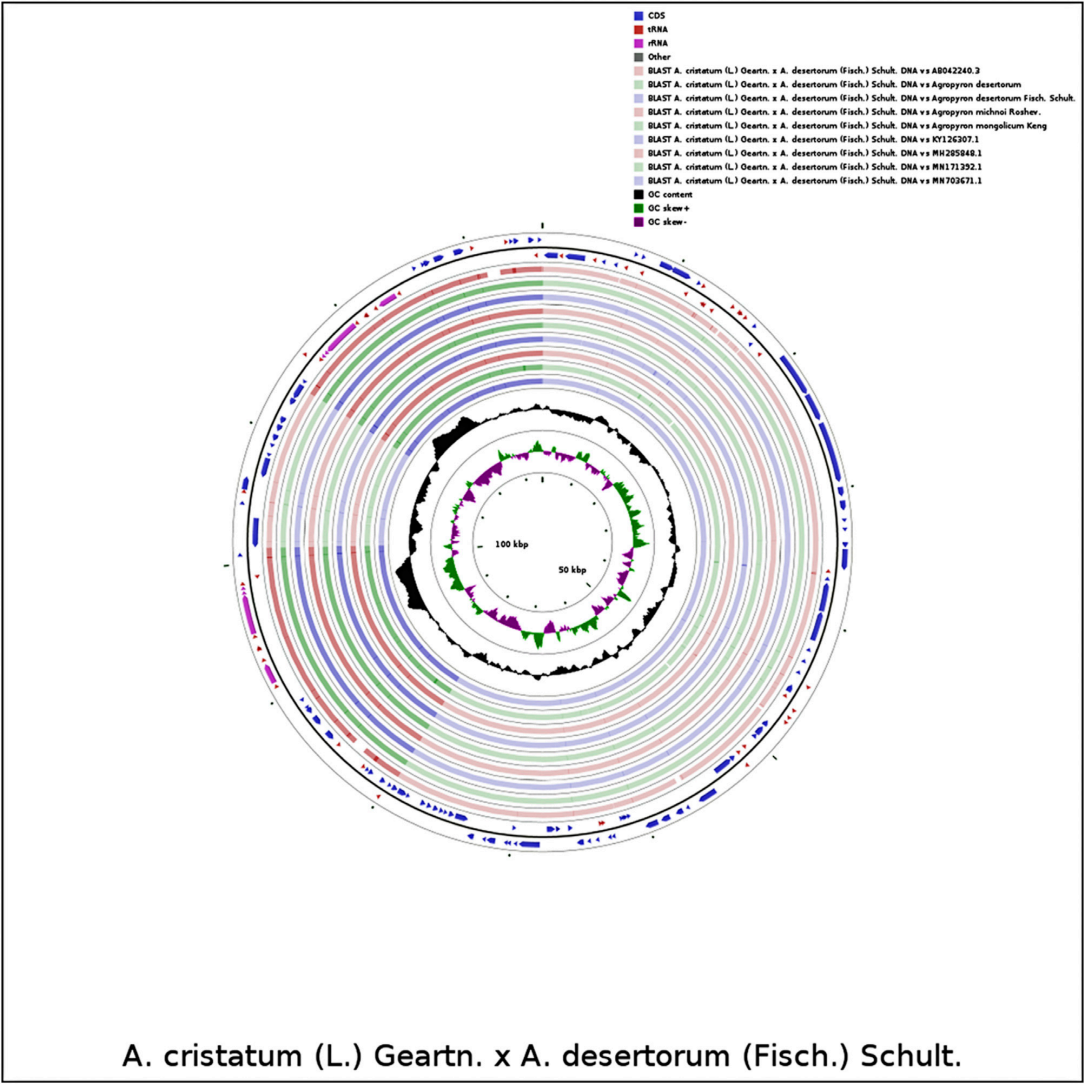
Comparative analysis of chloroplast genome structures. The two outermost circles describe the length and direction of genes in the genome; the circles inside represent similar results compared with other reference genomes; the black circles represent the GC content.

We compared the chloroplast genome of *A. cristatum* × *A. desertorum* Fisch. Schult. with other nine species, using the software CGVIEW (https://stothard.afns.ualberta.ca/cgviewserver). The default parameters were used for the comparative analysis of chloroplast genome structures for proximal species. Our results showed that the structures of the species of *Agropyron* are similar (Figure 7).

### Analysis of the IR/SC Boundary

**FIGURE 8:**
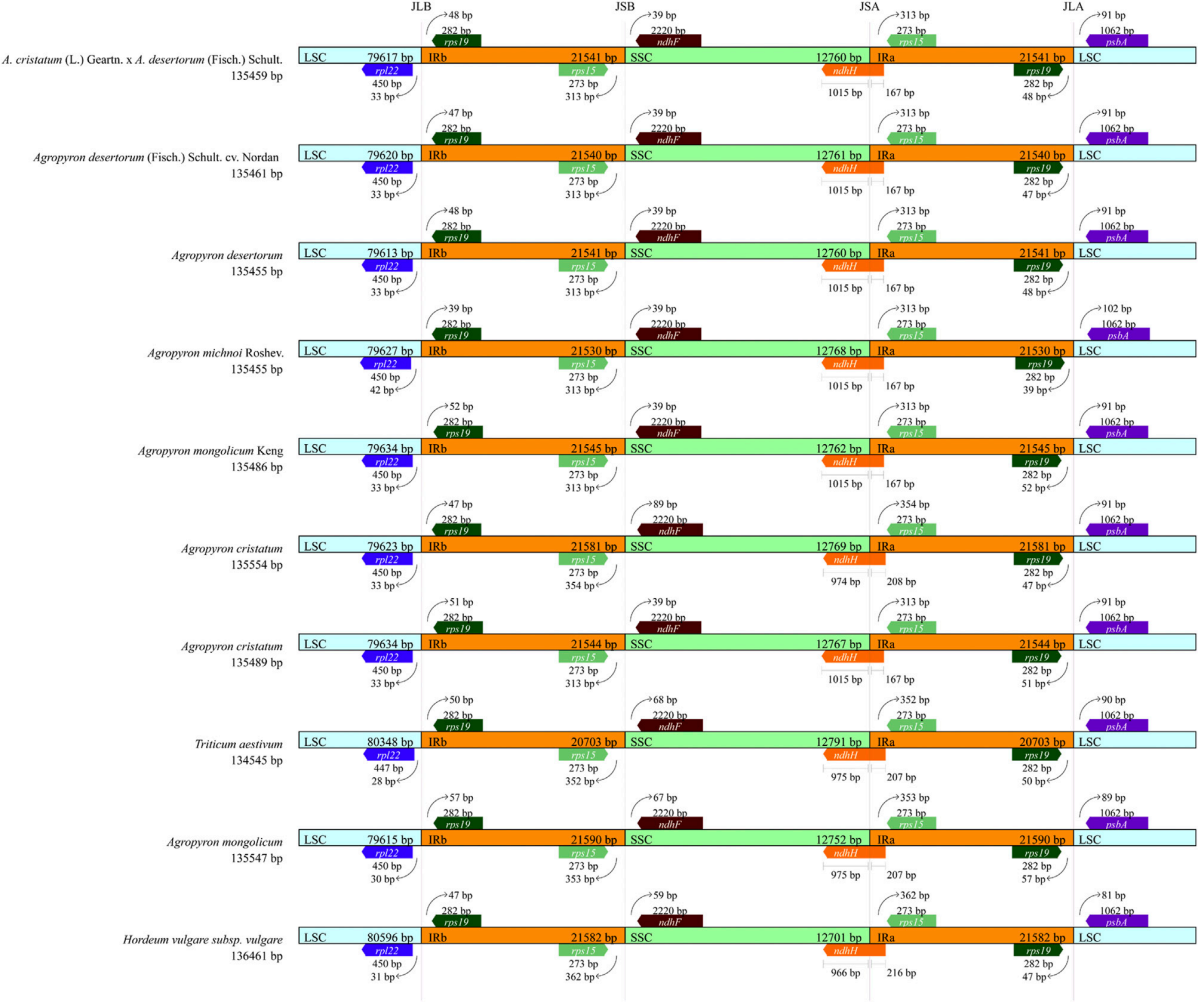
Analysis of IR boundary changes of chloroplast genomes. The thin lines represent the connection points of each region, and the map shows information about the genes near the connection points.

The chloroplast genomic DNA of *Agropyron* is a two-link molecule, and its size, structure, and gene capacity are highly conserved within four regions (LSC, SSC, IRa, and IRb). We compared the conserved four regions of ten species from the tribe *Triticeae*, including wheat, barley, and other eight species from the *Agropyron* genus (Figure 8). In comparison, the chloroplast genome of wheat is slightly smaller than that of *Agropyron*, whereas the chloroplast genome of barley is larger than that of *Agropyron* (Figure 8). In both wheat and barley, the LSC region is longer than that of *Agropyron*. The IR regions of the wheat chloroplast genome are shorter than those of the other nine species. The gene content and arrangement in the 10 species are the same in the IR region, which contain the *rps*19 and *rps*15 genes. In Figure 8, the *rps*19 gene is located in JLB (junction of LSC/IR) at 39 bp to 57 bp, and *rps*15 has 313 bp to 362 bp extending to JSB (junction of SSC/IRb). The *ndhH* gene spans the JSA (junction of SSC/IRA) region, and its length also reflects changes in the JSA region. The length distribution in six *Agropyron* species is the same. The *ndhF* gene is deviated from the JSB (IRB/SSC) region in the species studied, ranging from 39 bp in six species to 89 bp in *A. cristatum* (135,489 bp). The *rpl*22 gene is deviated from the JLB (IRB/LSC) region, ranging from 33 bp to 42 bp.

### Phylogenetic Analysis

**FIGURE 9:**
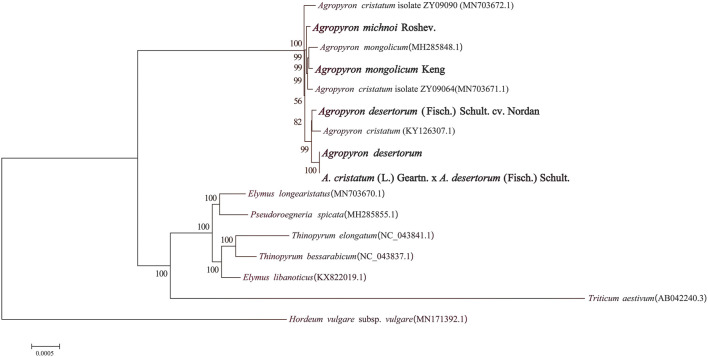
Phylogenetic tree of five species of *Agropyron* and related taxa based on the complete chloroplast genomes.

We selected 16 species in different genera from *Triticeae* and generated a phylogenetic tree, including nine species from *Agropyron*, two from *Elymus*, two from *Triticum*, one from *Roegneria* C. Koch, one from *Hordeum* Linn, and one from *Thinopyrum* (Figure 9). It was clear that these species were divided into two clades: the *Hordeum* genus is clustered in one clade, and all others are clustered in another clade (Figure 9). The species of the *Agropyron* genus were grouped in one subclade, while species from *Elymus*, *Pseudoroegneria*, *Thinopyrum*, and *Triticum* are in the other subclade. *A. mongolicun* Keng is close to *A. michnoi*, which is consistent with the structure and the SSR of these two species. *A. desertorum* Fisch. Schult, *A. desertorum*, and *A. cristatum* × *A. desertorum* Fisch. Schult. are in the same group (Figure 9). *A. desertorum* is more close to *A. cristatum* × *A. desertorum* Fisch. Schult, because the latter is a hybrid generated by crossing *A. desertorum* with *A. cristatum*.

## Discussion

### Characteristics of the Chloroplast Genomes of *Agropyron* Species

The objective of this study is to investigate the relationships among *Agropyron* species using chloroplast genome sequences. Similar to other plant species, the chloroplast genomes of *Agropyron* species show a four-segmented structure, consisting of a large single-copy region, a small single-copy region, and inverted repeat regions. *Agropyron* is closely related to *Triticum* genus, and its chloroplast genome features are similar to that of *Triticum* as well (Middleton et al., 2014). The sizes of *Agropyron* chloroplast genomes are comparable to previously reported complete chloroplast genomes of many angiosperms (Qian et al., 2013). Generally, the chloroplast genome size of angiosperms ranges from 72 to 217 kb. Although *A. cristatum* is close to *A. desertorum* Fisch. Schult. in the phylogenetic relationship, their gene numbers are different.

Generally, the common repeat type is the mono-nucleotide repeat, followed by the di-, tri-, and tetra-repeats, but penta-nucleotide repeats are not common. SSRs detected in chloroplast genomes of *Agropyron* are highly conserved in types and numbers, and most mono-nucleotide repeats in chloroplast genomes of *Agropyron* comprise base A or T, which is similar as reported in other plant species (Li et al., 2019). The SSRs are ideal genetic markers in plant molecular studies (Khan et al., 2019), and the SSRs are not completely uniform in *Agropyron*; thus, they can be used in the investigations of genetic diversity and genetic structure of *Agropyron*.

### Comparative Analysis of Chloroplast Genomes

During the evolution, contraction, and expansion of the IR, LSC and SSC regions are the main reason that leads to the size difference in chloroplast genomes (Yao et al., 2015). In this study, the difference of IR and SC regions of chloroplast genomes of *Agropyron* ranges from 33 bp to 313 bp. The *ndhH* gene crosses the JSA, which was also observed in other species of the *Triticeae* genus, such as *H. vulgare*, *B. vulgaris* (Chen et al., 2018), and *Festuca* (Qiu et al., 2019). The *ndhH* gene is sensitive to high light stress, which may have changed dramatically during evolution of land plants (Peng et al., 2011). The *ycf*1 gene crosses the JSA, which is also common in other families and genera, such as Fagaceae (Liu et al., 2019), *Liliales* (Nguyen et al., 2015), Zingiberaceae (Gui et al., 2020), and Anacardiaceae (Wang et al., 2020). As indicated by the Ka/Ks value and nucleotide diversity, changes in the *rpoC*2 gene in *A. michnoi* are greater than other species of *Agropyron* than the other four species and *A. cristatum* (KY 126307). Similarly, the Ka/Ks ratio of *ccsA* in *A. mongolicum* is greater than other species, except *A. michnoi*. For all the species of the *Agropyron* genus, the value of Ka/Ks is less than 0.5, indicating they are under purifying selection.

Based on the nucleotide diversity, we discovered the highly variable gene region *trnG-GCC*, which might be potentially informative molecular markers for species characterization of *Agropyron*.

### Phylogenetic Relationship of *Agropyron*

Chloroplast genomes have large amounts of genetic information and are easier than nuclear genomes for filtrating the single copy gene (Zhang and Li, 2011). So chloroplast genomes are widely used in plant evolutionary biology. Our analyses revealed that *A. michnoi* is close to *A. mongolicum* and that the polymorphic sites are rich in chloroplast genomes of *Agropyron*, including the mono-nucleotide site, indels, and SSRs (Figure 5). Plants of the *Agropyron* genus consist of the P genomes (Dewey, 1984). Plants with the P genome can be used to improve agronomic traits such as drought, cold, and disease resistance in wheat and other crops (Dong et al., 1992). Meanwhile, the P genome can also be used for species classification; for example, *A. cristatum* and *A. mongolicum* are found to be two species with significant genetic differentiation when the P genomes were analyzed. This point was also supported by the phylogenetic tree with the chloroplast genome that these two species were close but still separated in the tree (Figure 9). It was shown that *A. desertorum* is most likely derived from *A. cristatum* and *A. mongolicum* (Figure 9). Meanwhile, the bred variety *A. desertorum* Fisch. Schult. is close to *A. desertorum*, but *A. cristatum* × *A. desertorum* Fisch. Schult shows a closer relationship with *A. desertorum* than with *A. desertorum* Fisch. Schult., indicating that the chloroplast genome of *A. desertorum* plays a dominant role during evolution.

## Conclusion

We sequenced and assembled chloroplast genomes of five species from the *Agropyron* genus and compared their sequence at multiple levels. We found that the chloroplast genomes of *Agropyron* were different in the length, gene structure, and GC content. These chloroplast genomes of *Agropyron* are comparable with chloroplast genomes of other species within *Triticeae* and provide more insights into chloroplast genome evolution within *Triticeae*. From the phylogenetic tree, we can infer that they have a common ancestor. Based on the comparative analysis of chloroplast genomes of *Agropyron*, the intergenic Ka/Ks ratios are less than 0.5, suggesting that species of *Agropyron* are under a purifying selection. Therefore, our study lays a foundation for better understanding of the chloroplast genomes of *Agropyron* in species evolution, genetics, and phylogenetic relationships.

## Data Availability

The datasets presented in this study can be found in online repositories. The names of the repository/repositories and accession number(s) can be found in the article/Supplementary Material.
